# Franco Basaglia at 100: Human Rights and the Transformation of Psychiatry

**DOI:** 10.2174/0117450179503660261005113147

**Published:** 2026-10-13

**Authors:** Natia Horato, Mauro G. Carta, Antonio E. Nardi

**Affiliations:** 1 Laboratory of History of Psychiatry, Neurology and Mental Health, Institute of Psychiatry, Federal University of Rio de Janeiro, Rio de Janeiro (RJ), Brazil; 2 Department of Medical Sciences and Public Health, University of Cagliari, Cagliari, Italy

**Keywords:** Franco Basaglia, The Basaglian reform, Franco Basaglia's centenary, Psychiatric reform, Mental health care, Human rights issues

## Abstract

Franco Basaglia's centenary, celebrated on March 11, 2024, invites reflection on the historical significance of his legacy. His work in defense of people with mental disorders represents his fight for the protection of human dignity and the value of life. Pejoratively called “the philosopher Basaglia,” he transformed psychiatric practices by pushing the boundaries of medicine to address profound human rights issues. Basaglia adopted an existential-analytical stance that underpinned his commitment to psychiatric reform and the management of mental health practice, inspiring changes in public policies around the world. This commemorative editorial presents a historical overview of Franco Basaglia's contributions to psychiatric reform and their implications for mental health care.

## 1 LIFE, CAREER, AND PHILOSOPHY OF BASAGLIA

Franco Basaglia (1924–1980) was born in Venice, Italy, a city with a remarkable cultural history. He grew up in a Europe devastated by World War II, an environment that shaped his worldview and increased his sensitivity to human rights violations [1]. After his classical studies, Basaglia entered the Faculty of Medicine at the University of Padua in 1943, graduating in 1948. He began specializing in nervous and mental illnesses in 1952, later obtaining his habilitation in psychiatry in 1958 [1]. His academic training coincided with intense philosophical and scientific debates in post-war Europe, a period in which Italy served as an important site of cultural and political confrontations [2].

Basaglia increasingly recognized the deficiencies of the Italian psychiatric system, especially the fact that almost one hundred thousand people were institutionalized in asylums in the early 1960s, a significant number when compared to data from other nations. Moved by the conviction that psychiatric institutions contributed to the worsening of mental disorders, he began to pave the way for the deinstitutionalization of patients [2]. However, even without dismissing the usefulness of the medical approach, his defiant stance towards traditional academic hierarchies created significant obstacles to his university career [2, 3].

In 1961, Basaglia took over the direction of the Psychiatric Hospital of Gorizia, where he was faced, for the first time, with the harsh reality of institutions that functioned mainly as confinement centers, and not as therapeutic spaces [2]. Initially, he considered leaving office, feeling that he lacked the necessary resources to navigate the situation. However, by taking advantage of contacts and professional relationships established at academic conferences, he was able to build a wide network of collaborators [4]. His interventions in the hospital included the prohibition of the indiscriminate use of electroconvulsive therapy and the extinction of physical restraints, measures that reinforced his commitment to the well-being of patients [4]. Due to friction with conservative public opinion, Basaglia ended up leaving Gorizia. In 1968, he was appointed director of the Psychiatric Hospital of Trieste, where he implemented innovative institutional policies and was a pioneer in the integration of patients into the community. From these initiatives, it was demonstrated that individuals, regardless of their socioeconomic origin, could receive adequate treatment, as long as they were not involuntarily confined to environments that aggravated their health condition (Fig. **1**) [4, 5].

## 2 THE BASAGLIAN REFORM

Basaglia initiated a profound transformation in the conduct of the practice of psychiatry throughout Italy. He maintained that mental illness should not be conceptualized as a legal offense or a condition that required institutional confinement, but rather as a complex human experience that requires care and therapeutic assistance [6]. Rejecting repressive interventions, he defended that patients be recognized as subjects with rights. His initiatives contradicted the traditional psychiatric model, which historically advocated the isolation and social exclusion of individuals considered dangerous [6]. In one of his most striking statements, Basaglia argued that institutionalization did not treat the patient, but served to mitigate social discomfort by marginalizing individuals perceived as different [1]. From his point of view, the psychiatric hospital functioned as an instrument to control behaviors and expressions that deviated from social standards [2].

By dismantling the abuses that occurred in psychiatric institutions, Basaglia proposed a model of care based on human dignity and respect for the person [7]. He introduced innovative therapeutic modalities in these institutions, such as artistic activities, demonstrating that patients could reconstruct their identity and reestablish social bonds, despite the challenges posed by severe mental disorders [8]. For Basaglia, the involvement of patients in cultural events promoted self-expression beyond clinical diagnoses. Such initiatives represented not only a therapeutic alternative, but a critique of conventional psychiatric practices that marginalized the individuality of patients [7].

Basaglia's pioneering reforms transformed Italian psychiatry and exerted a significant influence on international mental health policy. On May 13, 1978, Law 180, known as the “Basaglia Law”, was enacted, which determined the progressive closure of psychiatric hospitals throughout Italy [8]. In addition, Basaglia profoundly inspired psychiatric reforms in Spain during the 1980s, encouraging policy reformulations in Western Europe and outlining models of community psychosocial rehabilitation throughout Latin America [9].

## 3 ITALY AFTER BASAGLIA

The disparity between the Basaglian Reform and contemporary practice is evident in institutions that historically opposed Basaglia’s ideas but now celebrate his legacy, even from a distorted and often critical perspective. In present-day Italy, there has been a considerable reduction in the availability of psychiatric treatment, as Italian mental health services face many challenges related to institutional practices, scarcity of resources, and an over-reliance on pharmacological interventions. Such aspects are considered by many scholars to represent a departure from Basaglia's original ideals [8].

This phenomenon is linked to the decentralization of health in Italy under the impact of economic austerity, but it manifests itself unevenly across different regions [10]. Financial constraints have disproportionately affected southern areas, generating substantial inequalities in the national implementation of primary mental health services and resulting in investments that remain well below the European average [10]. Although historical developments have led to a reduction in psychiatric hospitalizations, challenges related to the infrastructure of psychiatric care centers persist. This scarcity of resources has reignited the scholarly debate about the management of severe mental disorders in the post-deinstitutionalization era and the legacy of Basaglia.

The absence of an inclusive care model, added to the challenges of incorporating advances into mental health treatments, suggests that although psychiatric reform has gained strength, it has not brought concrete improvements to the population [8]. The medical community has not been able to consolidate a culture focused on efficient psychiatric interventions. In addition, the reduction in the public budget allocated to mental health and the clinical setback demonstrate a distancing from Basaglia's reform and the government's lack of interest in the most vulnerable populations [8].

## 4 FINAL REFLECTIONS

Despite the divergences regarding the principles that guided his reform, Franco Basaglia emphasized the premise that true progress in psychiatry does not depend only on diagnostic categories or technical interventions, but on the preservation of dignity and respect for the individual who experiences psychic suffering. Thus, Basaglia's fight for humanized psychiatric treatment remains essential, especially in a world where the stigma around mental illness is still evident.

## Figures and Tables

**Fig. (1):**
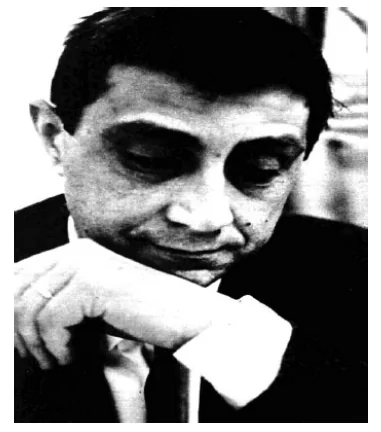
Franco Basaglia (1924 – 1980). Reproduced from Wikimedia Commons, Public Domain. “*The psychiatrist seems to discover only now that the first step towards the patient's healing is the return to the freedom that he himself, until now, had deprived him of” [5].*
